# Overnight extubation and risk of extubation failure in patients in the pediatric intensive care unit: an exploratory review

**DOI:** 10.3389/fped.2025.1700380

**Published:** 2025-11-21

**Authors:** Harold Andrés Payán-Salcedo, Valentina Ortiz Sandoval, Bivian Dairovy Castro Ibarguen, Yina Lisbeth Ramírez, Sergio Leonardo Cortés González, Leonardo Arzayus-Patiño

**Affiliations:** 1 Facultad de Salud, Programa de Fisioterapia, Universidad Santiago de Cali, Cali, Colombia; 2 Grupo de Investigación Salud y Movimiento, Universidad Santiago de Cali, Cali, Colombia

**Keywords:** pediatric intensive care units, tracheal extubation, intratracheal intubation, artificial respiration, extubation

## Abstract

**Background/objectives:**

Extubation in pediatric intensive care units (PICUs) requires care and thoroughness to minimize risks of extubation failure, systemic complications, and mortality. Up to 20% of patients in the PICU experience extubation failure, resulting in reintubation, longer hospital stays, and higher healthcare costs. Currently, no reviews have synthesized findings on the effect of overnight extubation and its association with extubation failure.

**Methods:**

This exploratory review was conducted based on the Joanna Briggs Institute methodology and PRISMA extension for scoping reviews guidelines. We analyzed studies collected from databases such as PubMed, Scopus, Web of Science, Science Direct, VHL Regional Portal, and Google Scholar using MeSH terms, Boolean operators, and a search strategy based on population, concept, and context (PCC). Studies that evaluated the impact of overnight extubation in the PICU were included, with the primary outcome being the association with the risk of extubation failure.

**Results:**

T Results: of 275 records identified, five studies met the inclusion criteria. Four studies found no significant association between overnight extubation and extubation failure, whereas one study with a larger sample reported a higher risk of reintubation during nighttime extubation. Overall, the findings revealed heterogeneous results influenced by population type, clinical context, and organizational factors.

**Conclusions:**

The available evidence does not consistently demonstrate that overnight extubation increases the risk of failure; however, some studies suggest that patient complexity and contextual factors may modify this relationship. Therefore, extubation decisions should be individualized according to clinical stability and available resources rather than based solely on the time of day, underscoring the need for prospective and standardized studies in this field.

## 1 Introduction

The pediatric intensive care unit (PICU) is a specialized area that aims to provide care to patients in critical condition or clinical instability, who are at a high risk of mortality and complications associated with their condition, requiring continuous monitoring and intensive multidisciplinary interventions (1, 2). Reportedly, up to 63% of pediatric patients often require mechanical ventilatory support for diagnoses such as sepsis, acute respiratory illness, and acute cardiorespiratory diseases (3). Hospitalization duration and resource use depend on the type of concurrent disease and disease history (4), which supports the importance of conducting arduous and relentless work toward an early and safe discharge of these patients.

Depending on its duration, mechanical ventilation can trigger a series of complications such as nosocomial infections, neuromuscular weakness due to the use of sedatives or relaxants for its coupling, delirium, iatrogenic drug withdrawal, pulmonary lesions, and ventilator-associated diaphragmatic atrophy (5–7). The latter develops approximately within 18 h after connection and progresses at an increasing rate of up to 2%–7% a day (8). This atrophy may result from a lack of effort by the patient, which subsequently decreases diaphragmatic contractility. Therefore, studies recommend maintaining adequate diaphragmatic activity and promoting continuous effort during mechanical ventilation to prevent diaphragm injury and deterioration (9, 10). Considering the above, mechanical ventilation weaning and minimized exposure should be continuously encouraged in pediatric patients. Efforts should not be spared in this regard, and it is essential to aim for extubation as soon as the patient's clinical condition allows (11).

Extubation of PICU patients is a crucial step that warrants thorough evaluation to ensure its effectiveness and safety and to avoid complications that may affect patients' health, such as extubation failure. Extubation failure is described as the inability to breathe spontaneously within the first 48 h after scheduled removal of the endotracheal tube, including the patient's difficulty in maintaining a patent airway during this period (12–14). Extubation failure occurs in approximately 4%–6% of patients in PICUs, with occurrences related to increased mortality rates and hospital costs (12, 13, 15). Different clinical approaches have been developed to determine the best time to perform extubation; however, to date, there is no clear consensus on the best technique or procedure to predict extubation failure. Overnight extubation has been commonly defined as the planned removal of the endotracheal tube between **19:00 and 07:00 h**, corresponding to the nighttime clinical shift. In the present review, this definition was adopted based on the criteria used by the included studies and with the aim of maintaining consistency with the existing literature, thereby ensuring comparability among the results.

The results of studies conducted on adult patients regarding the safety of overnight extubation and the risk of reintubation are heterogeneous and sometimes conflicting. Retrospective cohort studies (8, 14) have reported that overnight extubation is not associated with increased rates of reintubation or mortality among adult patients admitted to intensive care unit (ICUs). Gershengorn et al. (16) reported that the adults in their sample who underwent overnight extubation had higher mortality rates than those who were extubated during the day. In the context of critically ill pediatric patients, no reviews have grouped and synthesized the available scientific evidence to conclude whether performing extubation at night is associated with extubation failure. Therefore, the objective of this review is to collect evidence on whether overnight extubation is associated with an increased risk of extubation failure in patients admitted to PICUs.

## 2 Materials and methods

### 2.1 Protocol

An exploratory review was conducted based on the methodology of the Joanna Briggs Institute (JBI) (17), which was initially devised by Arksey and O'Malley (18) and the PRISMA extension for scoping reviews guidelines (19). To map the related literature, we conducted the following: defined precise inclusion and exclusion criteria, provided detailed participants' characteristics, selected a concept to guide the scope and depth of the review, and defined a context that limits the factors involved in the research.

### 2.2 Eligibility criteria

**Figure 1:**
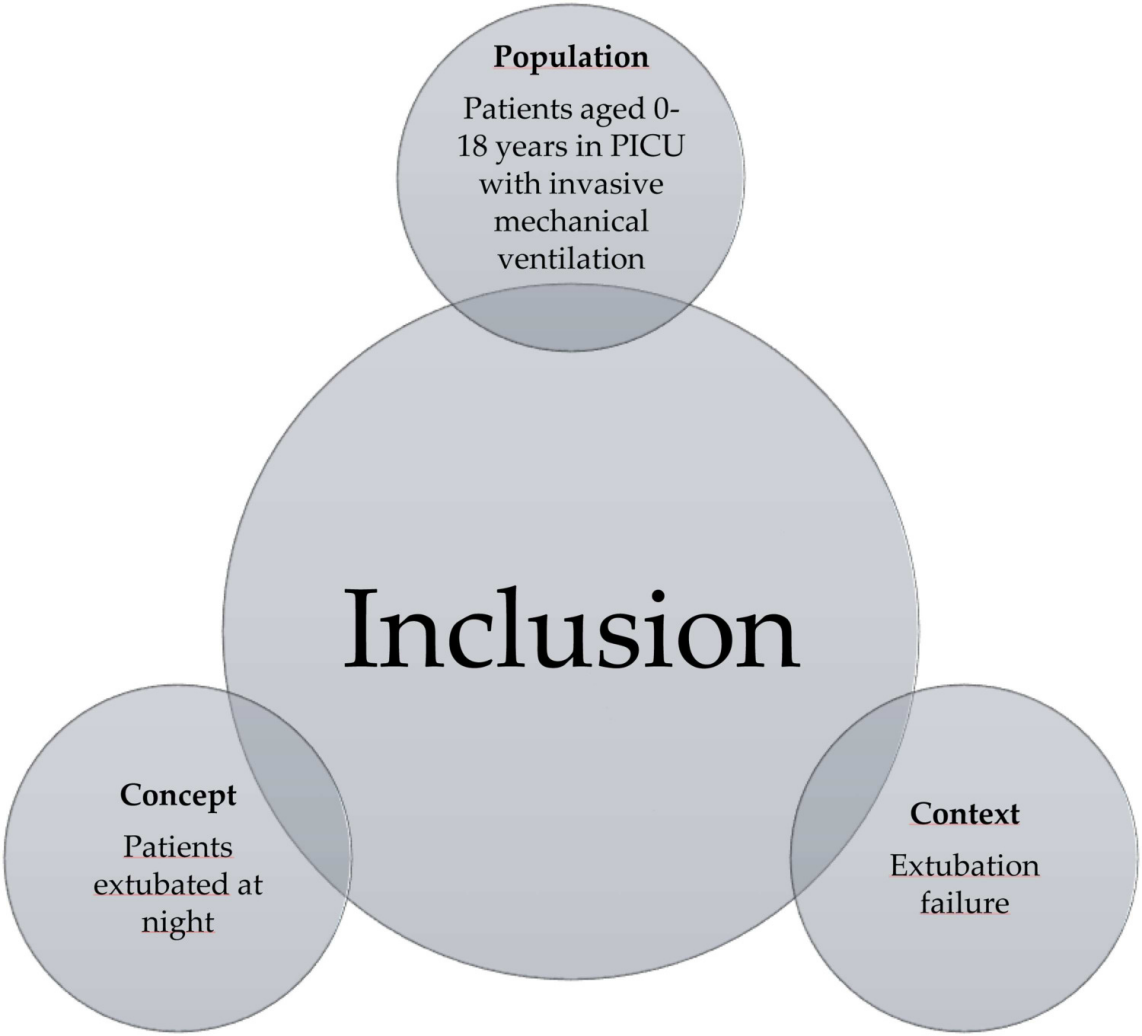
Inclusion criteria based on the PCC question.

To guide the review, a PCC question (17) was developed (Figure 1) including three elements: Population (P), Concept (C), and Context (C). According to the population (P), (P): We included studies that enrolled patients aged 0–17 years admitted to pediatric intensive care units (PICUs), including pediatric cardiac intensive care units (PCICUs) and neonatal intensive care units (NICUs), who received invasive mechanical ventilation. Based on concept (C), studies that performed extubated nights based on their methodology were included. For the purposes of this review, overnight extubation was defined as the planned removal of the endotracheal tube between **19:00 and 07:00 h**, consistent with the operational definitions used in the included studies and previous literature. At the Context level (C), we included studies that described extubation failure as one of their outcome measures. In terms of source types, experimental and quasi-experimental study designs were included, including randomized and nonrandomized controlled clinical trials, descriptive or analytical observational studies, and case reports/series.

### 2.3 Data sources

In line with JBI guidelines (17), a systematic and structured literature search was conducted in PubMed, Scopus, Web of Science, Science Direct, VHL Regional Portal, and Google Scholar databases in November 2024.

### 2.4 Search strategy

A search was performed in the abovementioned databases, using MeSH terms connected with Boolean operators. Additionally, a search Thesaurus (Table 1) was established following a PCC-based question structure to identify studies on whether overnight extubation is associated with the risk of extubation failure in patients admitted to PICUs.

**Table 1:** MeSH terms based on the PCC strategy.

PCC	Population	Concept	Context
MeSH	Intensive Care Units pediatric cardiac intensive care units neonatal intensive care units	Clinical practice patterns; Overnight extubation	Extubation failure

The search was conducted in English and included studies, without time or language limits.

### 2.5 Selection of studies/sources of evidence

**Figure 2:**
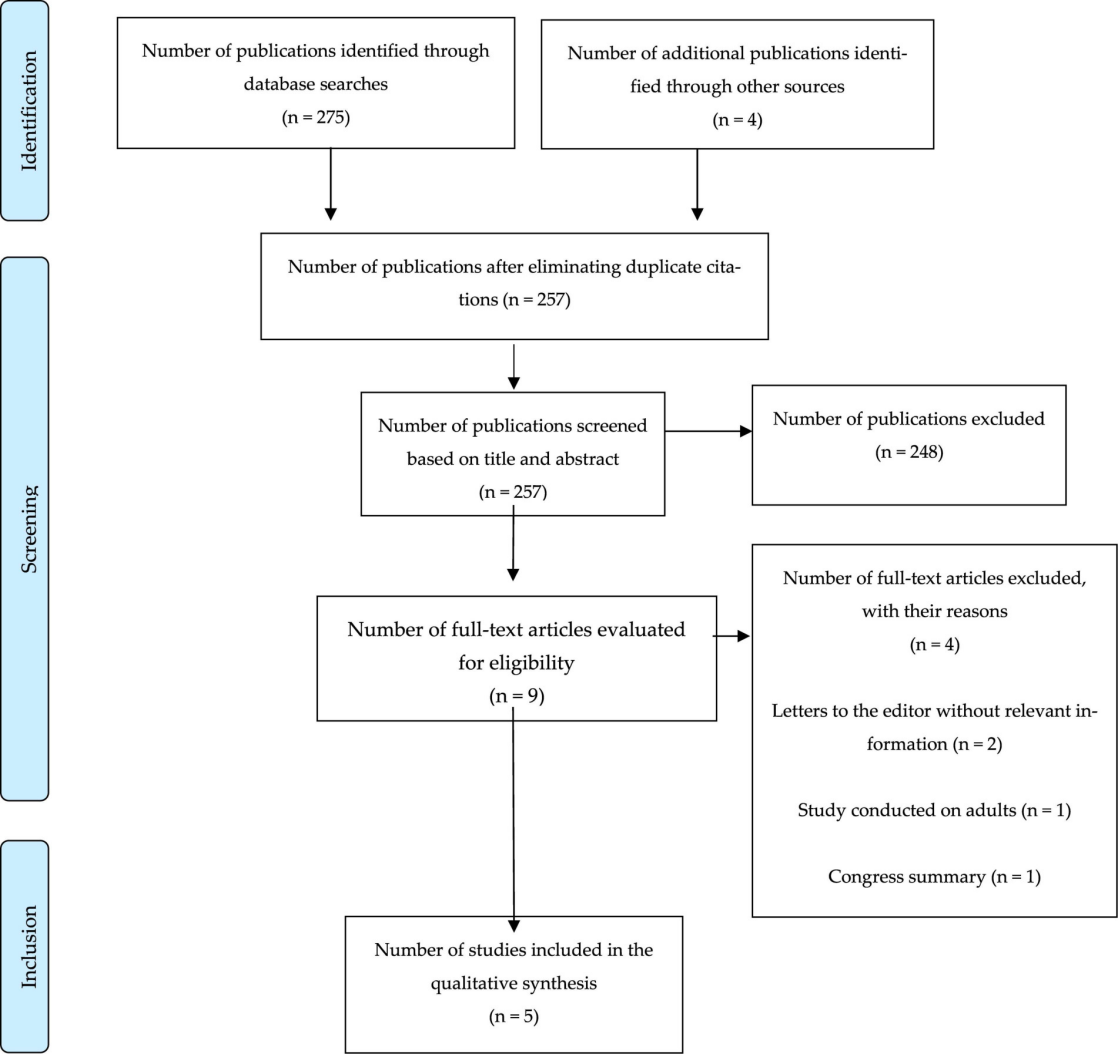
Item selection flowchart (figure PRISMA).

After the search, all citations that were identified were loaded into Mendeley version 2.98.0 2023 software (Mendeley Reference Manager, LDN, UK) and duplicate studies were removed. Two authors independently screened the articles' titles and abstracts and examined them based on the inclusion criteria to identify studies that had to be read thoroughly. Subsequently, these two independent reviewers, who were aware about the exclusion criteria, thoroughly evaluated the relevant full-text studies. Any disagreement was resolved by discussion or the inclusion of a third reviewer. The final outcome of the search and the inclusion/exclusion of studies was reported in a flowchart of reporting elements for systematic reviews and meta-analyses for the scoping review extension (19) (Figure 2). The reference list of all evidence sources was thoroughly examined to identify additional relevant studies through hand searching.

### 2.6 Data extraction and synthesis

An Excel spreadsheet was developed to extract data from the studies. The two reviewers manually and carefully extracted data from each of the databases to ensure data consistency and accuracy and avoid losing relevant information. Data extracted included specific details on authors, country, type of study, age of participants, sample size, definition of overnight extubation time, comorbidities, type of ICU, extubation protocols, duration on mechanical ventilation, length of ICU stay, and other key findings relevant to the development of the review.

### 2.7 Assessment of methodological quality

The methodological quality of the included studies was assessed using the Newcastle-Ottawa Scale (NOS), recommended by the Cochrane Collaboration for retrospective and prospective observational cohort studies. This scale assesses three domains: participant selection (maximum 4 points), comparability between groups (maximum 2 points) and outcome assessment (maximum 3 points), with a total score of 9 points. According to this score, the quality of the studies was classified as low (0–2 points), fair (3–5 points), and high (6–9 points) (20).

## 3 Results

The initial search of the databases identified 275 studies, 4 of which were identified by manual search through bibliographic references. 22 studies were excluded because they were duplicates, 248 after reading the title and abstract and 4 for not meeting inclusion criteria. Finally, five studies were included in the qualitative analysis (Figure 1). In terms of geographic distribution, 60% of the studies were conducted in the United States of America, 20% in Brazil and 20% in Chile. Regarding the methodological design, 100% were cohort studies, four were retrospective studies (8, 21–23), and one was a prospective study (24). The studies were published between 2016 and 2023.

Although its distribution was heterogeneous, the total sample size was 19,838 patients, ranging from newborns to 17-year-old children (Table 2). Overnight extubation times ranged from 19:00 to 08:00 h. Regarding extubation failure (requirement for reintubation <48 h of being extubated), values of patient who required overnight extubation ranged 3.8%–25%, with no statistically significant differences in most studies (8, 21, 22, 24) compared with those extubated during the day, with the exception of one study (23) that reported a significantly higher risk in overnight extubation (OR = 1.58, *p* = 0.02). This study (23) explicitly reported the type of ventilatory support used prior to extubation, whereas only two studies (8, 24) reported the use of a standardized protocol to guide the extubation process (Table 3).

**Table 2:** Characteristics of the studies included.

Authors	Country	Type of study	*n* (sample)	Age (weeks, months, or years) Mean (SD) Median [IQR]	Comorbidities	Unit type
Loberger et al. (8)	USA	Retrospective cohort study	555 patients D: 397 Ov: 185	D: 40 [9–114] months Ov: 38 [9–107] months	Status epilepticus, surgical problems	Medical-surgical PICU
Ibarra et al. (21)	Chile	Retrospective cohort study	146 patients D: 120 Ov: 26	O: 1.14 [0.25–5.5] years D: 1.14 [0.25–5.54] years Ov: 1.1 [0.15–4.25] years	Respiratory, neurological, and sepsis	Medical-surgical PICU
Guy et al. (22)	USA	Retrospective cohort study	379 patients D: 303 Ov: 76	D: 6.6 (0.5) weeks Ov: 6.7 (0.6) weeks	Low birth weight infants	PICU (neonatal) in three centers
Byrnes et al. (23)	USA	Retrospective cohort study	18,278 newborn patients: 4,130 Infants: 9,437 Children: 4,711	Newborns: 0–30 days old Infants: 31 days–1 year Children 1–18 years old	Airway abnormality, chromosomal abnormality	Postoperative PICU for cardiac surgery
Da Silva et al. (24)	Brazil	Prospective cohort study	480 patients D: 346 Ov: 134	O: 9 [3–48] months D: 8 [2–41] months Ov: 14 [3–60] months	Sepsis, cardiovascular diseases	General PICU

D, daytime extubation; O, overall population; Ov, overnight extubation; EF, extubation failure; PICU, pediatric intensive care unit; SD, standard deviation; IQR, interquartile range.

**Table 3:** Results of interest of the included studies.

Authors	Overnight extubation time	MV duration in days or hours Mean (SD)s Median [IQR]	PICU stay in days Mean (SD) Median [IQR]	Ventilatory support prior to extubation	Extubation protocol	Extubation failure (<48 h)	*p* value
Loberger et al. (8)	19:00 to 07:00	D: 62.5 [23–137.5] hours Ov: 37.6 [9.7–85] hours	D: 4.5 [1.9–9.0] Ov: 2.8 [0.9–6.5]	NR	Institutional ventilatory release pathway led by therapists and physicians, including daily assessment tool	D: 10.3% Ov: 8.1%	0.4
Ibarra et al. (21)	20:00 to 08:00	D: 10 (2) days Ov: 7 (1) days	D: 7 [3,75–14] Ov: 8 [5–14,75]	NR	NR	D: 5% Ov: 3.8%	0.8
Guy et al. (22)	19:00 to 07:00	D: 9.0 [14.1] days Ov:4.5 [8.9] days	NR	NR	NR	D: 24% Ov: 25%	0.6
Byrnes et al. (23)	20:00 to 6:00	0.96 [0.42–2.92] days	EF group: 24 [12–48] SE group 8 [5–14]	NR	NR	EF risk in the night shift relative to the day shift was significantly higher at 1.58 (1.07–2.3)	0.02*
Da Silva et al. (24)	20:00 to 08:00	D: 6 [4–11] days Ov: 5 [3–10] days	D: 13 [8–21] OV:11 [6–18]	CPAP 5 cmH_2_O or PSV with low parameters	Clinical evaluation + spontaneous breathing test under CPAP 5 cmH_2_O or PSV depending on tube caliber	D: 9.5% Ov: 10.4%	0.76

D, daytime extubation; O, overall population; Ov, overnight extubation; EF, extubation failure; EF group, group with extubation failure; SE group, successful extubation group; NR, not reported; CPAP, positive end-expiratory pressure; PICU, pediatric intensive care unit; PSV, pressure support ventilation; SD, standard deviation; IQR, interquartile range.

* Statistically significant difference.

The comorbidities of the population assessed varied: in 40% of the investigations (21, 24), patients presented respiratory diseases, sepsis and cardiovascular diseases, whereas in the remaining 60% (8, 22, 23), those with low birth weight, status epilepticus, airway anomalies, and chromosomal abnormalities were included. Regarding the duration of mechanical ventilation, on average, it ranged from 0.96 to 7 days in patients extubated at night and from 62.5 h to 10 days in patients extubated during the day. Similarly, ICU stay ranged from 2.8 to 11 days in patients extubated at night and from 4.5 to 13 days in patients extubated during the day.

Regarding the methodological evaluation of the studies conducted using the NOS for observational studies, three of the five studies analyzed were of high quality (8, 23, 24), whereas the remaining two were classified as moderate quality (score: 6/9) (21, 22). Studies with the highest methodological quality were characterized as adequate cohort representativeness, clarity in the definition of exposure, and adequate assessment of the outcome. Conversely, limitations in the moderate-quality studies included limited intergroup comparability, absence of a clearly defined unexposed group, and lack of information on loss to follow-up (Supplementary Table S1).

## 4 Discussion

The objective of this review was to map the existing literature to explore whether overnight extubation is associated with an increased risk of extubation failure in critically ill pediatric patients. In general, most of the included studies (8, 21, 22, 24) reported no significant differences between daytime and overnight extubation, suggesting that the time of day would not have a determining influence on outcomes. However, one large sample study (23) did find a statistically significant association between overnight extubation and increased risk of reintubation. This discrepancy suggests that the impact of extubation time could depend on the clinical context, patient profile, and organizational conditions of each unit. Thus, further studies with comparable designs are required to establish more solid conclusions.

This finding can be interpreted considering several factors. First, the sample size of the study was considerably larger than in other studies (*n* = 18,278), which provided greater statistical power to detect intergroup differences and a broader representation of pediatric patients. However, it is important to note that the population consisted mainly of postoperative cardiac surgery patients, many of whom underwent prolonged and complex procedures, including congenital heart defect repairs and transplants. These characteristics may have contributed to delayed extubations occurring during nighttime hours, reflecting surgical and recovery dynamics rather than planned overnight extubations. This context should be considered when interpreting the association between extubation timing and extubation failure reported in that study.

This volume of data also implies that even small changes or modest effects can reach statistical significance, which may not be observable in studies with smaller samples. Additionally, the population included newborns, infants, and children up to 18 years of age, most of whom had complex comorbidities like airway anomalies and chromosomal alterations, which alone increase the risk of extubation failure (25), especially in less controlled clinical settings.

Similarly, the authors of the study (23) indicated that the increased risk observed could be related to organizational factors, such as scheduling extubations outside regular working hours or the reduced availability of specialized staff during the night. These conditions may indirectly influence clinical outcomes more than the timing itself. However, the absence of a standardized extubation protocol and the lack of information regarding the ventilatory mode prior to support withdrawal limit the ability to establish causal relationships. Overall, the findings suggest that although most studies did not show significant differences, the results of Byrnes et al. (23) highlight the importance of basing extubation decisions on objective and context-specific clinical criteria, avoiding generalizations and prioritizing patient safety.

However, given that there are gaps in pediatric literature regarding the analysis of variables of interest that may influence postextubation outcomes, it is important to analyze the evidence available in adults as an initial reference point to guide the discussion and generation of conclusions. In studies about critically ill adult populations, the results are still contradictory and very similar to those shown in our review. Research such as those of Krebs et al. (14), Tischenkel et al. (26) and Everhart et al. (27) indicate that overnight extubation does not increase mortality or the risk of reintubation, even in patients with associated comorbidities. Moreover, Gershengorn et al. (16) in their cohort study of 97,844 ICU patients, were able to demonstrate that those extubated at night had higher rates of ICU and in-hospital mortality than those extubated during the day.

Consistently with the results of the studies included, which did not show an increase in extubation failure when extubation was performed at night (8, 21, 22, 24), Patel et al. (28) conducted a retrospective cohort study in 680 adult patients, 280 of whom underwent overnight extubation between 19:00 and 6:59, and were able to determine that overnight extubations did not present higher risks of mortality or reintubation and reported benefits such as shorter duration of mechanical ventilation and shorter hospital stay. It is important to mention that, due to differences attributed to morphophysiology, diseases, and ventilation and support strategies, these results in adults cannot be extrapolated to the pediatric population. Therefore, further research is needed to guide decision making.

Extubation practices in pediatric intensive care units (PICUs) differ substantially from those in adult ICUs due to differences in physiology, disease spectrum, and organizational structure. In PICUs, extubation decisions are generally based on a multidisciplinary assessment that integrates respiratory mechanics, neurological function, and hemodynamic stability rather than fixed protocolized thresholds. Institutional policies and team composition during the night shift may play a decisive role in determining whether extubation is performed. In units where cardiovascular surgeries are performed, it is relatively common for postoperative patients to return from the operating room intubated but to be extubated within the first 12 h—regardless of the time of day—once hemodynamic and bleeding control have been confirmed. Conversely, patients with acute respiratory distress syndrome, complex comorbidities, or difficult airways require more cautious evaluation, as extubation in these cases largely depends on the availability of experienced personnel and adequate monitoring. These clinical and organizational differences highlight that the safety and timing of extubation in pediatric populations are determined not only by physiological readiness but also by the resources and operational protocols of each unit.

The reluctance of healthcare personnel to perform extubations during the night shift in ICUs is explained by multiple factors that influence clinical decision making. A research by Balas et al. (29) identified that, in adults, postponing extubation until daytime, a common practice of night teams, is a significant cause of delays. This is partly attributed to the fact that night-shift staff prefer to wait for evaluation by more experienced physicians and to the perception that they may not feel completely comfortable making extubation decisions without adequate supervision. Additionally, conducting spontaneous breathing tests at night, when fewer resources and personnel are available, raises safety concerns as any complications could be more difficult to manage in that setting (26).

Despite the fact that overnight extubation has not been consistently associated with an increased risk of failure, there is still considerable reluctance on this regard in pediatric clinical practice. This trend may be driven by organizational and risk perception factors rather than clinical evidence. A study conducted by Mekonnen et al. (30) discussed the causes of unplanned extubation in pediatric patients, identifying patients' agitation, inadequate endotracheal tube fixation, and nursing staff workload as factors associated with unplanned extubation. Additionally, there were no significant differences between day and night shifts regarding these outcomes. These findings reinforce the idea that many of the adverse events related to extubation are associated with other conditions occurring in the hospital setting, not exclusively with the time of extubation.

The findings of this review suggest that the timing of extubation should not be determined solely by the time of day but rather by a comprehensive assessment that considers both clinical and contextual factors. Decisions should be guided by patient stability, ventilatory support requirements, comorbidities, available unit resources, and the clinical judgment of the healthcare team, which may include performing overnight extubation under reasonable circumstances. In this regard, some studies conducted in pediatric patients (31, 32) have highlighted the importance of considering additional factors such as patient condition, ventilatory support, and comorbidities when predicting extubation success. Although the available evidence does not allow for a definitive conclusion that overnight extubation is entirely safe or equivalent to daytime extubation, it also does not support postponing it solely based on timing. Despite advances in the management of mechanical ventilation and extubation in pediatric patients, there is still limited scientific evidence specifically describing the impact of extubation timing on outcomes in PICUs (33). Therefore, additional clinical studies should be developed to broaden the current research context and guide decision-making in this field.

During nighttime hours, organizational factors such as a reduced physician-to-patient ratio and limited availability of certified intensivists outside regular working hours may influence adverse clinical outcomes in PICUs. Gupta et al. (34) reinforced this concern in their multicenter study, demonstrating that continuous intensivist presence (24 h a day, 7 days a week) is associated with significantly lower mortality and improved clinical outcomes in critically ill pediatric patients, particularly in complex cases requiring immediate decisions such as extubation. This situation could indirectly contribute to the suboptimal use of hospital resources, as decisions such as postponing extubation may unnecessarily prolong mechanical ventilation and ICU stay (35). In this regard, the implementation of standardized, evidence-based protocols could enhance the quality of pediatric intensive care, reduce costs, and optimize the allocation of specialized staff according to care demands and patient safety (31, 36).

Taken together, the findings of this review indicate that, although there is no definitive consensus regarding the impact of extubation timing on clinical outcomes, the available evidence does not consistently suggest that the time of day is a determining factor. Accordingly, extubation decisions should be guided by objective clinical criteria, taking into account patient stability, resolution of the underlying cause of mechanical ventilation, required support, and the characteristics of the care environment. Significant gaps remain in the pediatric literature concerning the influence of overnight extubation on the risk of extubation failure. Moreover, while evidence from adult studies provides valuable context, it cannot be directly extrapolated to pediatric populations. Therefore, prospective multicenter studies using standardized weaning and extubation methodologies—including organizational, clinical, and ventilatory variables are needed to better understand the factors that truly determine extubation success or failure in PICUs.

Regarding the methodological quality of the studies, most presented a favorable evaluation according to the NOS's criteria, used for retrospective and prospective observational cohort studies. Three studies were classified as high quality, whereas two were classified as moderate quality. The main methodological strengths of these studies included adequate representativeness of the cohorts, clarity in determining exposure, and consistent definition of the main outcome. Although two studies were of moderate quality, the methodological level of the evidence included was good in general terms, which increases the robustness of our findings. Nevertheless, results should be interpreted with a sustained critical and analytical approach, considering that all the studies are observational, and do not allow direct causal relationships. Therefore, our findings should be interpreted with caution, taking into account the limitations of the retrospective design and the heterogeneity of the clinical characteristics and protocols used.

### 4.1 Strengths

One of the main strengths of this review was the rigorous application of checklists and standardized protocols for the systematic search in multiple databases, which allowed an exhaustive and structured mapping of the available literature on the subject. Similarly, the evaluation of the methodological quality of the studies strengthens the interpretation of the findings. To our knowledge, this is the first review to collect, analyze, and synthesize the results of studies that have evaluated the possible association between extubation time and risk of extubation failure in PICUs. This work is an important starting point for future research and is a call to multidisciplinary PICU teams to develop more comprehensive and methodologically robust studies that help to strengthen evidence-based clinical decision making.

### 4.2 Limitations

Although this review provides valuable information on the possible relationship between overnight extubation and the risk of extubation failure in PICUs, there are some limitations that should be considered. Most of the studies were retrospective and observational, which implies an inherent risk of bias in patient selection, data collection, and interpretation of results. Additionally, heterogeneity in the criteria used to define eligibility for extubation, previous ventilation methods, and application of standardized protocols as well as differences in available resources and clinical settings, may have influenced the variability of the reported findings.

Another relevant aspect is the wide age range covered by the “pediatric patient” category, which includes newborns and adolescents aged 18 years. This diversity in developmental stages, pathophysiological conditions, ventilatory needs and clinical response to respiratory support could have differentially impacted the outcomes analyzed, making it difficult to generalize results and apply them uniformly to the entire pediatric population. In this regard, future research should prioritize more rigorous designs, ideally clinical trials or prospective multicenter studies, that allow a more accurate assessment of the impact of extubation times. Such studies should also consider additional variables such as baseline disease, type of previous ventilatory support, specific age group, and the participation of multidisciplinary teams in clinical decision making.

## 5 Conclusions

Based on the studies reviewed, no direct association can be established between the timing of extubation and the risk of extubation failure in critically ill pediatric patients. The available evidence shows heterogeneous results, and although most studies did not identify a significant increase in risk associated with overnight extubation, this finding should be interpreted with caution. Decisions regarding the optimal timing of extubation should be individualized according to patient condition, case complexity, and the resources available within each unit. Additional prospective and multicenter studies are needed to generate more robust and generalizable conclusions.
