# Supplementary material for: Overnight extubation and risk of extubation failure in patients in the pediatric intensive care unit: an exploratory review
Source: Front Pediatr. 2025 Nov 21;13:1700380. doi: 10.3389/fped.2025.1700380 (PMC12678291; doi:10.3389/fped.2025.1700380)
Supplement: Supplementary file 1 [file Table1.docx]

**Supplementary table 1.** Assessment of methodological quality of included studies (Newcastle-Ottawa scale)

| **Authors** | **Representativeness of the exposed cohort** | **Selection of the unexposed group** | **Determination of exposure** | **Absence of outcome at baseline** | **Comparability of cohorts** | **Assessment of outcome** | **Adequate follow-up** | **Acceptable loss to follow-up** | **Total score (out of 9)** |
| --- | --- | --- | --- | --- | --- | --- | --- | --- | --- |
| Loberger et al., 2021 (5) | * | * | * | * | ** | * | * | * | 9/9 |
| Byrnes et al., 2023 (18) | * | * | * | * | * | * | * | * | 9/9 |
| Da Silva et al., 2016 (19) | * | * | * | * | * | * | * | * | 8/9 |
| Guy et al., 2021 (17) | * | - | * | * | * | * | - | - | 6/9 |
| Ibarra et al., 2023 (16) | * | - | * | * | * | * | - | - | 6/9 |
